# Minor papilla approach improves technical success of nasopancreatic drainage-based pancreatic juice cytology for early pancreatic cancer diagnosis

**DOI:** 10.1055/a-2631-7957

**Published:** 2025-07-23

**Authors:** Tatsunori Satoh, Shinya Kawaguchi, Haruna Takahashi, Yuichi Masui, Masanori Matsuda, Asami Kawai, Shinya Endo, Takafumi Kurokami, Naofumi Shirane, Kazuya Ohno

**Affiliations:** 1 26389 Gastroenterology, Shizuoka General Hospital, Shizuoka, Japan

**Keywords:** Pancreatobiliary (ERCP/PTCD), Diagnostic ERC, ERC topics, Quality and logistical aspects, Performance and complications

## Abstract

**Background and study aims:**

Early detection of pancreatic cancer (PC) is vital for improving survival, yet it often relies on indirect imaging findings rather than detection of distinct masses. Recently, pancreatic juice cytology obtained via nasopancreatic drainage (NPD-PJC) has emerged as a valuable diagnostic approach for early-stage disease. However, technical challenges associated with NPD placement remain a significant limitation. This study aimed to assess whether incorporating a minor papilla approach improves the technical success rate for NPD-PJC in patients with suspected early-stage PC.

**Patients and methods:**

We conducted a retrospective study of patients scheduled for NPD placement for NPD-PJC between January 2015 and November 2024. Demographic and procedural data were collected, including endoscopic retrograde pancreatography (ERP) findings and outcomes associated with major and minor papilla approaches. Potential risk factors for technical failure and post-ERP pancreatitis (PEP) were evaluated.

**Results:**

A total of 81 cases were planned for NPD-PJC within the study period to differentiate early-stage PC. The success rate of the major papilla approach alone was 81.5%, which significantly increased to 93.8% ( *P* = 0.00157) with the addition of the minor papilla approach. Abnormal ductal configurations were associated with failure of the conventional approach (odds ratio 23.4). The minor papilla approach was not a significant risk factor for PEP, whereas younger age (≤ 70 years) and high body mass index (≥ 25) were identified as PEP risk factors.

**Conclusions:**

Incorporating the minor papilla approach substantially improves technical success of NPD-PJC without increasing PEP risk, underscoring the importance of individualized ERP strategies for early-stage PC diagnosis.

## Introduction

Pancreatic cancer (PC) remains one of the most lethal malignancies worldwide, characterized by a high incidence, elevated mortality, and poor prognosis 1 2 . These unfavorable outcomes are largely attributable to late-stage diagnoses, with only a small fraction of patients identified at a surgically resectable stage 3 4 5 . Consequently, early detection is paramount to improving survival 3 . Recent studies on early PC detection have underscored the importance of indirect indicators, such as main pancreatic duct (MPD) dilation in the absence of a detectable mass 4 6 7 8 9 10 11 . Additional emphasis has been placed on pancreatic parenchymal atrophy (PPA) and hypoechoic regions adjacent to MPD strictures observed on endoscopic ultrasonography (EUS) 8 11 12 13 .

Endoscopic ultrasound-guided tissue acquisition (EUS-TA) is effective for pathological confirmation in cases where a visible tumor is present 14 15 . However, in cases featuring only indirect signs, pancreatic juice cytology (PJC) obtained via endoscopic nasopancreatic drainage (NPD-PJC)—commonly referred to as serial pancreatic juice aspiration cytologic examination (SPACE)—serves as a pivotal diagnostic approach 7 16 17 18 19 20 . NPD-PJC is particularly valuable for identifying PC, including early-stage lesions, even when tumors are not readily visible 20 21 .

A growing body of literature has documented the diagnostic utility of NPD-PJC 7 16 17 18 19 20 21 22 , but most studies focus on diagnostic accuracy in patients who successfully undergo NPD tube placement via the major papilla. Anatomical variations in the pancreatic duct, however, are well-recognized 23 and can complicate NPD placement. For example, in the context of pancreatic stone treatment, cases involving complex ductal anatomy often preclude a major papilla approach and necessitate a minor papilla approach, which has been reported to be effective 24 25 . By extension, in patients with suspected early-stage PC undergoing NPD-PJC, a subset may similarly be unsuitable for a major papilla approach, potentially necessitating the use of the minor papilla 26 . Despite this, few studies have addressed technical nuances of NPD placement, including the minor papilla approach, in patients with suspected early-stage PC.

We, therefore, hypothesized that supplementing the conventional major papilla route with an optional minor papilla approach would increase the technical success rate for NPD placement for NPD-PJC. However, concerns remain that the minor papilla approach may be associated with a higher risk of post-endoscopic retrograde cholangiopancreatography (ERCP) pancreatitis (PEP). Accordingly, the present study aimed to investigate the technical success rate of NPD tube placement (SPACE) incorporating a minor papilla approach and to explore potential strategies for improving its feasibility in patients suspected of having early-stage PC.

## Patients and methods

### Study design and patients

This retrospective, single-center, observational study included patients who were scheduled for NPD tube placement for NPD-PJC between January 2015 and November 2024. In cases in which NPD-PJC was performed multiple times during the study period, clinical information and ERCP findings from the initial NPD-PJC session were used for analysis. All data were retrieved from hospital medical records and endoscopic databases.

The study protocol was approved by the Institutional Review Board of Shizuoka General Hospital (approval number: SGHIRB#2024042), and the investigation was conducted in accordance with the Declaration of Helsinki. Given the retrospective nature of the study, the requirement for informed consent was waived.

### Definitions

#### Patient characteristics

Age, body mass index (BMI), and laboratory values were based on measurements obtained at or near the time of ECRP. A smoking history was defined as having smoked more than 100 cigarettes within 1 month or having been a habitual smoker for ≥ 6 months at any point. A drinking history was defined as consuming at least one alcoholic drink per week for ≥ 1 year. A history of acute pancreatitis was defined as prior hospitalization for acute pancreatitis before the first ERCP.

Pancreatic duct morphology and stenosis of the MPD were evaluated using magnetic resonance cholangiopancreatography (MRCP) and ERCP images. Based on recent studies, MPD morphology was categorized as normal, reverse-Z subtype, loop subtype, or divisum 23 27 28 . Two board-certified gastroenterologists in pancreatology (TS and SK) independently evaluated these imaging findings.

Technical failure was defined as unsuccessful NPD tube placement, regardless of whether the approach was via the major or minor papilla. Technical failure of the conventional approach was defined as unsuccessful NPD tube placement via the major papilla.

#### Indication for serial pancreatic juice aspiration cytologic examination

At our hospital, NPD-PJC is indicated for cases with indirect findings suggestive of PC on imaging (CT, MRI, or EUS), despite absence of a clearly identifiable mass. It is also considered in cases in which a mass is detectable but ≤ 10 mm in size, making EUS-TA challenging, or when EUS-TA fails to obtain malignant findings. Based on previous reports, indirect findings in PC are defined as including MPD stenosis or dilatation 6 16 29 , localized PPA 8 9 10 11 , and hypoechoic areas surrounding MPD stenosis 4 6 7 10 .

#### Endoscopic retrograde cholangiopancreatography

ERCP was performed using conventional lateral-view endoscopes. Pancreatography was initially attempted via the major papilla with a standard catheter. Under the supervision of an expert (defined as having performed ≥ 400 ERCP procedures), a non-expert operator could perform the procedure. Once pancreatography was obtained, the guidewire and catheter were advanced deeply into the MPD (near the presumed lesion), followed by NPD tube placement. If the MPD could not be visualized on ERP from the major papilla or if the guidewire failed to advance into the dorsal pancreatic duct, the minor papilla approach was attempted.

If a non-expert operator was unable to achieve pancreatic duct cannulation within approximately 10 minutes, the procedure was handed over to an expert. For patients strongly suspected of having pancreatic duct anomalies on preprocedural MRCP (e.g., complete pancreatic divisum), a minor papilla approach was attempted early. Otherwise, the decision and timing for transitioning to a minor papilla approach were left to the expert’s discretion after an initial attempt via the major papilla.

Rectal diclofenac suppositories were routinely administered as standard prophylaxis for PEP. Intravenous ulinastatin and aggressive periprocedural hydration were not routinely used. In cases in which NPD placement was unsuccessful, pancreatic stents were not placed for PEP prevention, because guidewire access to the pancreatic duct was typically not achieved.

#### Minor papilla cannulation technique

**Fig. 1:**
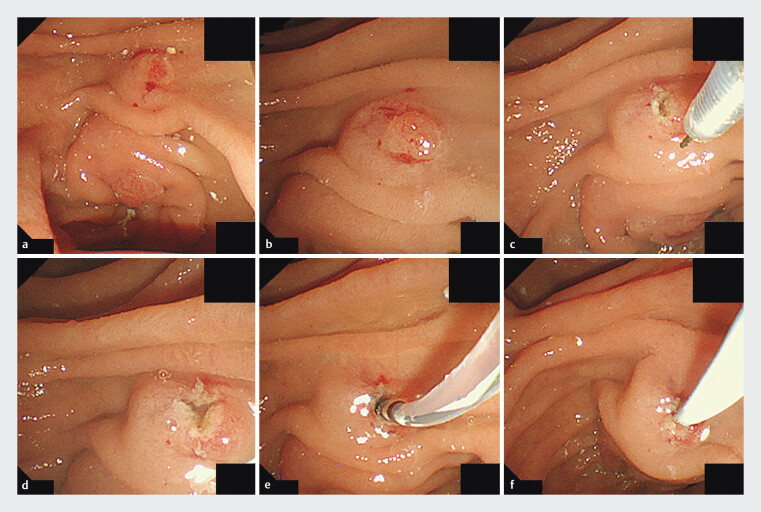
Minor papilla cannulation technique with precut. Endoscopic view illustrating minor papilla cannulation using a needle-knife precut technique. A three-radial star-shaped incision was made to expose the orifice and facilitate guidewire and catheter advancement for NPD tube placement.

The minor papilla was first identified endoscopically ( Fig. 1 **a** , Fig. 1 **b** ). If it could not be visualized at all, the approach via the minor papilla was abandoned. Cannulation was attempted using an MTW catheter in combination with a 0.025-inch guidewire. When pancreatography through the minor papilla was successfully achieved and the guidewire could be advanced into the MPD, the NPD tube was placed via this route. If the guidewire could traverse the minor papilla but the catheter could not, a tapered catheter (PR-110Q; Olympus Medical Systems) was used. If even the tapered catheter could not be inserted, a minor papilla incision was performed to facilitate tube placement. This was done using a needle-knife (KD-10Q-1; Olympus Medical Systems) in a precut manner, creating a three-radial star-shaped incision ( Fig. 1 **c** , Fig. 1 **d** ), following the method described in a prior case report 26 . The catheter was then advanced and the NPD tube placed ( Fig. 1 **e** , Fig. 1 **f** ). If MPD cannulation through the minor papilla ultimately failed, the procedure was terminated.

#### Nasopancreatic drainage tube placement and pancreatic juice cytology

Once the NPD tube was placed, it remained in situ overnight, and pancreatic juice was collected approximately six times over 2 days, including the day of ERCP. Fresh intraductal pancreatic juice was obtained directly through the NPD tube at each sampling timepoint; any accumulated fluid in the external collection bottle was discarded to minimize risk of cellular degradation. Cytological examination was performed on each fresh sample. Secretin was not administered during these procedures. Cases in which the planned number of samples could not be obtained owing to accidental tube dislodgement or other technical issues were classified as failures after NPD placement. PJC was evaluated collaboratively by a cytotechnologist and a pathologist. Cytological findings were categorized into three groups: positive for malignancy, suspicious for malignancy, and negative. For the purposes of this study, any case in which pancreatic juice cytology yielded a result of “positive” or “suspicious” for PC on at least one occasion was defined as NPD-PJC-positive.

#### Final diagnosis

Final diagnosis of malignancy was established based on either surgical pathology or clinical course during a minimum 12-month follow-up period. In resected cases, histopathological evaluation of pancreatic specimens was performed, with high-grade PanIN (PanIN-3) and invasive PC classified as malignant according to the 8th edition of the Union for International Cancer Control classification. For nonsurgical patients with suspected PC, imaging follow-up was conducted for at least 1 year. Cases without evidence of disease progression during this period were considered non-malignant.

#### Adverse event-related ERP and NPD placement

PEP was defined as newly developed abdominal pain accompanied by elevated serum pancreatic amylase to at least three times the upper limit of normal within 24 hours after ERCP. PEP severity was graded according to Cotton’s criteria 30 . Other adverse events (AEs) were diagnosed and classified in accordance with the American Society for Gastrointestinal Endoscopy lexicon for endoscopic AEs 31 .

#### Study outcomes

The primary outcome of this study was to evaluate whether incorporating a minor papilla approach improves the success rate for NPD tube placement after an unsuccessful major papilla approach. In addition, patient characteristics were compared between the conventional approach, minor papilla approach, and technical failure groups to identify potential factors affecting success of NPD placement. Furthermore, the study aimed to assess incidence of and risk factors for post-ERCP AEs, particularly those associated with the minor papilla approach.

#### Statistical analysis

Continuous variables are presented as medians with interquartile ranges (IQRs) and were analyzed using either the Mann-Whitney U test or Student’s *t* -test, as appropriate. Categorical variables are expressed as frequencies (percentages) and were compared using Fisher’s exact test. The McNemar test was performed to evaluate whether the success rate of NPD tube placement improved by attempting the minor papilla approach after an unsuccessful major papilla approach. Univariate logistic regression was performed to identify predictive factors of PEP and technical failure, reporting odds ratios (ORs) and 95% confidence intervals (CIs). For continuous variables, a receiver operating characteristic curve was generated to determine the cutoff value. Variables with *P* < 0.20 in the univariate analysis were included in a multivariate model. All tests were two-tailed, and *P* < 0.05 was considered significant. Statistical analyses were conducted using R version 3.4.1 (The R Foundation for Statistical Computing, Vienna, Austria).

## Results

### Study population and baseline characteristics

A total of 81 patients were included in this study. Baseline demographic and clinical characteristics are presented in Table 1 . Median age of the cohort was 75 years, and 50.6% of patients were men. Median BMI was 21.78. MPD stenosis was present in 85.2% of cases, with a median maximum duct diameter of 3.0 mm. Regarding pancreatic duct morphology, 77.8% of patients had a normal MPD configuration, 11.1% had a reverse-Z subtype, 3.7% exhibited a loop subtype, and 7.4% were diagnosed with pancreatic divisum.

**Table:** **Table 1** Study population and baseline characteristics.

Number of patients	81
Age, years, median (IQR)	75.00 (69.00, 78.00)
Sex, man, n (%)	41 (50.6)
BMI, median (IQR)	21.78 (9.64, 23.85)
Drinking history, yes, n (%)	28 (34.6)
Smoking history, yes, n (%)	30 (37.0)
Diabetes mellitus, yes, n (%)	22 (27.2)
AMY, median (IQR)	95.0 (72.0, 134.0)
CEA, median (IQR)	2.45 (1.65, 3.23)
CA19–9, median (IQR)	11.0 (6.75, 20.0)
Site of target lesion, Ph/Pbt, n (%)	12 (14.8)/ 69 (85.2)
MPD stenosis, yes, n (%)	69 (85.2)
Maximum MPD diameter, median (IQR)	3.0 (3.0, 4.0)
MPD morphology	
Normal, yes, n (%)	63 (77.8)
Reverse Z, yes, n (%)	9 (11.1)
Loop, yes, n (%)	3 (3.7)
Divisum, yes, n (%)	6 (7.4)
AMY, amylase; BMI, body mass index; CA19–9, carbohydrate antigen 19–9; CEA, carcinoembryonic antigen; IQR, interquartile range; MPD, main pancreatic duct; Ph, pancreas head; Pbt, pancreas body or tail.	

### Procedure outcome and adverse events

**Fig. 2:**
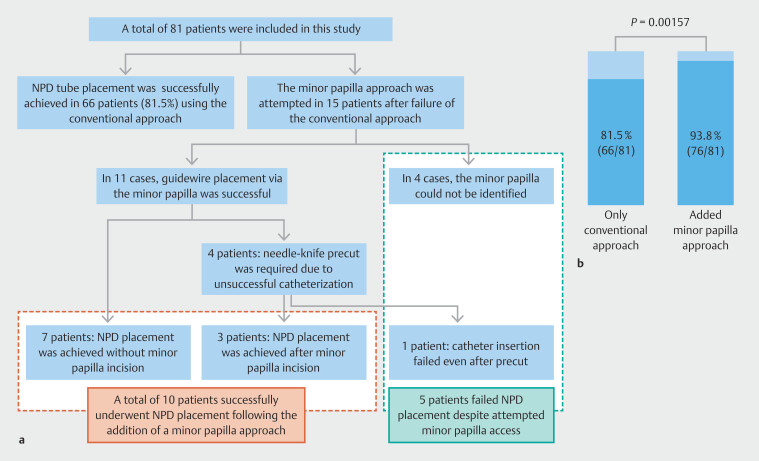
Technical success flow and success rate of nasopancreatic drainage tube placement: conventional approach versus the added benefit of the minor papilla approach. The NPD placement rate was 81.5% with the conventional approach. By incorporating the minor papilla approach for unsuccessful cases, the overall nasopancreatic drainage placement rate significantly increased to 93.8% ( *P* = 0.00157).

Procedure-related data are summarized in Table 2 . Median procedure duration for ERCP and subsequent interventions was 15.0 minutes. The technical success flow is illustrated in Fig. 2 **a** . Among the 15 patients in whom the minor papilla approach was attempted, the minor papilla could not be endoscopically identified in four cases, and the procedures were terminated. All four of these patients had a normal-type MPD configuration. In the remaining 11 patients, NPD tube placement was successfully achieved in seven cases without the need for additional intervention. In the other four cases, a needle-knife precut technique was employed to facilitate cannulation; of these, three resulted in successful NPD placement, whereas one case failed due to inability to advance the catheter despite precut. No patients in this cohort required endoscopic pancreatic sphincterotomy or the rendezvous technique. The conventional approach alone was successful in 81.5% of cases (66/81). When the minor papilla approach was attempted following failure of the major papilla approach, the overall success rate improved significantly to 93.8% (76/81) (McNemar test, *P* = 0.00157) ( Fig. 2 **b** ). Median duration of NPD placement was 2 days, with a median of six PJC samples obtained per patient. PEP occurred in seven patients (8.6%), of whom five had mild disease and two had moderate disease. One patient (1.2%) developed cholangitis, and three (3.7%) experienced inadvertent NPD tube dislodgement.

**Table:** **Table 2** Procedure outcome and adverse events.

Number of patients	81
Procedure time, minutes, median (IQR)	15.0 (12.00, 24.00)
Overall success rate of NPD tube placement, n (%)	76 (93.8)
Conventional method, n (%)	66 (81.5)
Adding minor papilla approach, n (%)	76 (93.8)
NPD size, F, median (IQR)	5 (4, 5)
Duration of NPD placement, days, median (IQR)	2 (2, 2)
Number of cytology samples, median (IQR)	6 (6, 6)
Post ERCP adverse events	
PEP, n (%)	7 (8.6)
Mild/moderate/severe	5 (6.2)/2 (2.5)/0 (0)
Cholangitis, yes, n (%)	1 (1.2)
Hemorrhage, yes, n (%)	0 (0)
Dislodgement, yes, n (%)	3 (3.7)
ERCP, endoscopic retrograde cholangiopancreatography; IQR, interquartile range; NPD, nasopancreatic drainage; PEP, post-ERCP pancreatitis	

### Comparison between major papilla approach success group and unsuccessful group

Table 3 compares patient characteristics between the major papilla approach successful group (n = 66) and the unsuccessful group (n = 15), which includes patients who required a minor papilla approach (regardless of outcome) and those in whom NPD placement ultimately failed. No statistically significant differences were observed in age, sex, BMI, or tumor markers between the groups. However, MPD morphology differed significantly ( *P* < 0.001), with a greater proportion of reverse‑Z and divisum types in the unsuccessful group. Procedure duration was also significantly longer in the unsuccessful group (30.0 minutes vs. 15.0 minutes, *P* < 0.001). AE rates were similar across both groups.

**Table:** **Table 3** Comparison between major papilla approach successful group and unsuccessful group.

	Successful conventional approach group	Unsuccessful conventional papilla approach group	
Number of patients	66	15	*P*
Age, years, median (IQR)	75 (69–78)	71 (70–78)	0.747
Sex, male, n (%)	33 (50.0)	8 (53.3)	1.000
BMI, median (IQR)	21.61 (19.76–23.81)	22.00 (19.22–24.37)	0.908
Drinking history, yes, n (%)	22 (33.3)	6 (40.0)	0.850
Smoking history, yes, n (%)	24 (36.4)	6 (40.0)	1.000
Diabetes mellitus, yes, n (%)	18 (27.3)	4 (26.7)	1.000
AMY, median (IQR)	95.5 (80.0–134.75)	79.00 (49.50–125.50)	0.319
CEA, median (IQR)	2.50 (1.70–3.40)	1.90 (1.45–2.70)	0.204
CA19–9, median (IQR)	11.00 (5.00–20.00)	10.00 (9.00–15.00)	0.748
Location of target lesion, Ph, n (%)	11 (16.7)	1 (6.7)	0.561
MPD stenosis, yes, n (%)	57 (86.4)	12 (80.0)	0.823
Maximum MPD diameter, median (IQR)	3.0 (3.0–4.0)	3.0 (2.0–4.5)	0. 849
MPD type			< 0.001
Normal, yes, n (%)	59 (89.4)	4 (26.7)	
Reverse Z, yes, n (%)	4 (6.1)	5 (33.3)	
Loop, yes, n (%)	3 (4.5)	0 (0.0)	
Divisum, yes, n (%)	0 (0.0)	6 (40.0)	
Procedure time, minutes, median (IQR)	15.0 (10.25–18.00)	30.00 (27.00–55.00)	< 0.001
Route of NPD placement, major/ minor/ unsuccess	66/0/0	0/10/5	
NPD size, Fr, median (IQR)*	5.00 (4.00–5.00)	5.00 (4.00–5.00)	1*
Duration of NPD placement, days, median (IQR)*	2.00 (2.00–2.00)	2.00 (2.00–2.00)	0.612*
Number of cytology samples, median (IQR)*	6.00 (6.00–6.00)	6.00 (6.00–6.00)	0.471*
Post ERCP adverse events			
PEP, yes (%)	5 (7.6)	2 (13.3)	0.836
Mild/moderate	3 (4.5)/ 2 (3.0)	2 (13.3)/ 0 (0)	-
Cholangitis, yes (%)	1 (1.5)	0 (0)	1.000
Hemorrhage, yes (%)	0 (0)	0 (0)	1.000
Dislodgement, yes (%)*	3 (4.5)	0 (0)	1.000*
AMY, amylase; BMI, body mass index; CA19–9, carbohydrate antigen 19–9; CEA, carcinoembryonic antigen; ERCP, endoscopic retrograde cholangiopancreatography; IQR, interquartile range; MPD, main pancreatic duct; NPD, nasopancreatic drainage; Ph, pancreas head; Pbt, pancreas body or tail; PEP, post-ERCP pancreatitis *Comparisons between the conventional approach group and the group in which NPD placement was ultimately achieved via the minor papilla.			

### Diagnostic yield of cytology via nasopancreatic drainage tube

Among the 76 patients in whom NPD tube placement was successfully achieved (66 via the major papilla and 10 via the minor papilla), four patients who had less than 12 months of follow-up without surgery were excluded. The remaining 72 cases (62 major papilla and 10 minor papilla) were evaluated for diagnostic performance of NPD-PJC using final clinical diagnosis as the reference standard. Of these, 41 patients (56.9%) were ultimately diagnosed with malignancy. The NPD-PJC results were positive in 36 patients (50.0%), including one false-positive case, and negative in 36 patients (50.0%).

Overall sensitivity, specificity, and diagnostic accuracy of NPD-PJC were 0.854 (95% CI 0.708–0.944), 0.968 (0.833–0.999), and 0.903 (0.810–0.960), respectively. In subgroup analysis, diagnostic performance in the major papilla group (n = 62) showed a sensitivity of 0.879 (0.718–0.966), specificity of 0.966 (0.822–0.999), and accuracy of 0.919 (0.822–0.973). In the minor papilla group (n = 10), sensitivity, specificity, and accuracy were 0.750 (0.349–0.968), 1.000 (0.094–1.000), and 0.800 (0.444–0.975), respectively.

### Risk factor analysis for conventional approach and technical failure and PEP

Potential risk factors for technical failure of the conventional approach are presented in Table 4 . Abnormal MPD configuration (reverse-Z, loop, or divisum) was strongly associated with failure (OR 23.2, *P* < 0.001 in the univariable model; OR 23.4, *P* < 0.001 in the multivariable model). Age, sex, and BMI did not have statistically significant effects on failure of the conventional approach.

**Table:** **Table 4** Univariable and multivariable logistic regression analysis for technical failure of conventional method.

Variable (reference)	Category	Univariable logistic model			Multivariable logistic		
		OR	95% CI	*P* value	OR	95% CI	*P* value
Age (> 70 years)	≤ 70 years	0.62	0.157–2.44	0.493			
Sex (female)	Male	1.14	0.372–3.51	0.816			
BMI (< 25)	≥ 25	1.64	0.444–6.03	0.459			
AMY (< 85 U/mL)	≥ 85 U/mL	0.41	0.131–1.28	0.123	0.40	0.0954–1.65	0.204
MPD stenosis (absence)	Presence	0.63	0.149–2.69	0.534			
Site of target lesion (Pbt)	Ph	0.36	0.0425–3.00	0.343			
MPD morphology (normal)	abnormal	23.2	5.79–92.8	< 0.001	23.4	5.65–97.0	< 0.001
BMI, body mass index; CI, confidence interval; MPD, main pancreatic duct; OR, odds ratio; Ph, pancreas head; Pbt, pancreas body or tail.							

Univariable and multivariable logistic regression analysis of PEP risk factors is shown in Table 5 . In univariate analysis, younger age (≤ 70 years; OR 14.2, p = 0.0168) and higher BMI (≥ 25; OR 6.89, *P* = 0.0195) were significantly associated with PEP. In the multivariate model, these two factors remained independently associated with PEP: age ≤ 70 years (OR 37.3, 95% CI 2.25–619.0; *P* = 0.0115) and BMI ≥ 25 (OR 24.6, 95% CI 2.13–284.0; *P* = 0.0103). Neither the minor papilla approach nor abnormal MPD morphology was significantly correlated with PEP incidence.

**Table:** **Table 5** Univariable and multivariable logistic regression analysis for post ERCP pancreatitis.

Variable (reference)	Category or unit	Univariable logistic model			Multivariable logistic		
		OR	95% CI	*P* value	OR	95% CI	*P* value
Age (> 70 years)	≤ 70 years	14.2	1.61–125	0.0168	37.3	2.25–619.0	0.0115
Sex (female)	Male	0.71	0.1490–3.4	0.669			
BMI (< 25)	≥ 25	6.89	1.36–34.8	0.0195	24.6	2.13–284.0	0.0103
AMY (< 85 U/mL)	≥ 85 U/mL	0.38	0.99–1.00	0.646			
MPD stenosis (absence)	Presence	1.05	0.115–9.57	0.967			
Procedure time (≤15 minutes)	> 15 minutes	2.79	0.508–15.3	0.238			
Minor papilla approach (absence)	Presence	1.20	0.13–11.2	0.870			
Success of conventional approach (yes)	No	1.88	0. 33–10.8	0.480			
MPD morphology (normal)	abnormal	2.95	0.60–14.6	0.185	2.14	0.262–17.5	0.478
BMI, body mass index; CI, confidence interval; MPD, main pancreatic duct; OR, odds ratio; Ph, pancreas head; Pbt, pancreas body or tail.							

## Discussion

This retrospective study of 81 patients undergoing NPD tube placement for NPD-PJC demonstrates that incorporating the minor papilla approach in addition to the conventional major papilla route significantly increased the overall technical success rate from 81.5% to 93.8%. These results underscore the importance of a flexible approach to ERCP, particularly in patients with anatomical variations of the pancreatic duct system. Although numerous studies have documented the diagnostic performance of NPD-PJC 7 16 17 18 19 20 , few have specifically focused on technical success of NPD tube placement. To the best of our knowledge, this is the first report to provide data on the general success rate for NPD tube placement and to demonstrate that including the minor papilla approach substantially increases overall success without increasing of risk of PEP.

These findings have important clinical implications for early detection and management of PC. Early-stage PC is notoriously difficult to diagnose because it often presents only with indirect signs (e.g., MPD stenosis, focal PPA) rather than a discrete mass 7 16 17 18 19 20 . NPD-PJC, which relies on repeated pancreatic juice cytology, has proven to be an invaluable technique for identifying both overt and subtle lesions of the pancreas 7 16 17 18 19 20 . The success of NPD-PJC, however, fundamentally depends on ability to place an NPD tube, and our results show that systematically including the minor papilla approach can substantially expand the indications for and success of this diagnostic method. Ultimately, the improved technical success rate may translate into more accurate diagnoses of early PC lesions, thus potentially improving patient outcomes through earlier therapeutic interventions.

Although diagnostic performance of NPD-PJC has been reported to be high for early-stage PC 17 18 19 21 , data specifically evaluating its utility via the minor papilla are scarce. In this study, we found that diagnostic yield of NPD-PJC via the minor papilla was comparable to that via the major papilla, despite the limited number of cases. Sensitivity was slightly lower in the minor papilla group (0.750 vs. 0.879), but specificity and accuracy remained high, with no false positives observed. These findings suggest that the minor papilla approach can still provide clinically valuable cytologic information when the major papilla route is not feasible. Notably, diagnostic performance was not significantly compromised, supporting its use as a technically feasible and reliable alternative. This approach may be particularly beneficial in patients with anatomical variations, such as pancreatic divisum, or in cases with difficult ductal access. While further validation is needed, our data support incorporation of the minor papilla approach into individualized diagnostic strategies for challenging cases.

Pancreatic duct anomalies are known to occur in a certain subset of patients 23 28 , often complicated NPD tube placement via the major papilla. Previous studies on pancreatic stone management have reported that minor papilla cannulation can be crucial for both diagnostic and therapeutic interventions in patients with pancreatic divisum or reverse-Z ductal anatomy 24 27 32 . Consistent with these findings, our study confirms that abnormal MPD configurations (reverse-Z, loop, or divisum) were strongly associated with failure of the conventional major papilla approach (OR 23.4), whereas subsequent application of a minor papilla approach frequently led to successful NPD tube placement. This suggests that consideration of the minor papilla route might be highly beneficial in patients with suspected ductal anomalies. However, in cases in which the minor papilla cannot be identified endoscopically, this approach is not feasible. Therefore, it may be advisable to assess visibility of the minor papilla before initiating cannulation via the major papilla. In patients in whom the minor papilla is clearly visible, proactively attempting a minor papilla approach—especially in the presence of suspected anatomical variation—may increase likelihood of successful NPD placement.

All 10 cases in which the minor papilla approach was used had no instances of tube dislodgement, and planned pancreatic juice collection was successfully performed, confirming that NPD-PJC can be effectively performed via the minor papilla. Both 4F and 5F NPD tubes were successfully placed in our cohort. However, from a technical standpoint, given the potentially smaller orifice of the minor papilla, using a thinner NPD tube may facilitate smoother placement.

PEP is a well-recognized complication of ERCP and is important to consider when performing NPD-PJC. Although concerns exist that a minor papilla approach may increase PEP risk, our data suggest otherwise—only one patient in the minor papilla cohort experienced PEP, with no significant difference compared with the results with conventional NPD placement. Furthermore, in our multivariate analysis, the minor papilla approach was not an independent risk factor for PEP. Although several risk factors for PEP have been described in the context of conventional ERCP 30 33 34 , data specifically addressing PEP risk during NPD-PJC—especially in cases of suspected carcinoma in situ—remain limited. Notably, we identified younger age (≤ 70 years) and high BMI (≥ 25) as risk factors for PEP in our cohort, which is consistent with previous studies implicating younger age and obesity as risk factors for PEP in standard ERCP settings 34 35 . Awareness of these risk factors is critical for optimizing patient selection and assessing risk when performing NPD-PJC, and larger, prospective investigations are warranted to further elucidate the precise risk profile for PEP after NPD tube placement for NPD-PJC in individuals with suspected early-stage PC.

This study has several limitations. First, the retrospective, single-center design may limit the generalizability of our findings. Second, the relatively small sample size of the minor papilla group (n = 10) and the technical failure group (n = 5) precludes definitive subgroup analyses. Third, although we identified potential risk factors for technical failure and PEP, causal relationships cannot be drawn owing to the study’s observational nature. Fourth, although we demonstrated improved technical success with NPD placement, its effect on diagnostic accuracy remains undetermined. Nevertheless, cytological outcomes in cases of suspected early-stage PC are unlikely to depend on the specific route of NPD placement, and ensuring technical success of placement itself is critical. Prospective, multicenter studies with standardized protocols are warranted to validate these results and further elucidate optimal integration of the minor papilla approach for early PC screening and diagnosis.

## Conclusions

Incorporating the minor papilla approach substantially improves the technical success of NPD-PJC without increasing PEP risk, underscoring the importance of individualized ERP strategies for early-stage PC diagnosis.

## Data Availability Statement

The data presented in this study are available on request from the corresponding author. The data are not publicly available owing to privacy concerns.
